# Diseases of the Mouth. Review of Dr. Sexton's Article

**Published:** 1880-02

**Authors:** 


### ARTICLE II.

### *S. Sexton, M. D., on Diseases of the Mouth.**

BY PROF. HODGKIN.—*Review.*

The frequency with which diseases of the oral cavity and more especially of the teeth, excite affections in parts more or less remote, has of late attracted the attention of medical men, (we use the term, as contradistinguished from dentists,) in a marked degree, and their knowledge of these subjects has been freshened, as the more candid of them readily acknowledge by the original researches of our specialty. It is well that the partition walls which in the past have separated medicine from dentistry are thus gradually thining, by a sort of process of absorbtion, the regular medical practitioner deriving some benefit, to say the least, from the humbler and possibly, slighted labors of his dental colaborer.

But it seems that while this is true in at least some degree, and that dentistry is possessed of some facts which medicine is glad to use, that in the main the knowledge of the principles which underlie the rational practice of dentistry, and a knowledge of the more prominent facts in dental pathology, are very obscurely understood as yet by medical men generally. We arise from a perusal of the article by Dr. Sexton on "affections of the Ear arising from Diseases of the Teeth," with the conviction that a medical man is not fully competent to criticise the work or the

*"On affections of the Ear arising from Diseases of the Teeth. S. Sexton, M. D., Surgeon to the New York Ear Dispensary," etc. —American Journal of Medical Sciences, Jan. 1880.

methods of dentistry; and indeed feel assured that no one but a daily worker in the mouth can well judge of the significance of the diseased conditions which there arise. And in the outstart we disclaim making any apology or defence of dentistry. Its faults are prominent enough, and the damaging character of the work of many of its practitioners is sufficient to call for as severe strictures as the writer of the article under review indulges in. Indeed it is well once in a while to read what outsiders think of us and our work. But the medical man must, of necessity be a sciolist in matters of dentistry, and such Dr. Sexton proves himself.

We have headed this as quoting the title of a paper on "diseases of the mouth." Such in fact the paper is; for while professedly a treatise on "affections of the Ear arising from diseases of teeth," the writer so soon abandons his subject and discusses at large the injuries which are done and may be done by injudicious or incompetent dentistry, as to justify the first title.

Still this is a monograph of value. There is no dental student but can profit by the introductory parts of it, treating of the phyisological relations between the ear and the teeth; and a careful study of the very rational pathology enunciated cannot but point to a more successful treatment of diseased conditions. The quotations from Woakes and Foster as to the association of the vaso-motor with the cerebro-spinal nerves, and his comments upon these, are exceedingly interesting. He thinks too, what seems possible, that even cases of "deaf muteism" may be caused in infancy by the irritant action of teething, and he quotes from Hilton on "Rest in Pain," one of the cheap series of books issued by Wm. Wood & Co. last year, on the characteristic ulcers of the tongue due to irritation by a jagged tooth.

But when Dr. Sexton comes to the description of the treatment of diseases of the teeth, he certainly betrays the lack of accurate knowledge of the subject—possibly possessing as much as most men outside the practice of dentistry—but assuredly not sufficient to justify the assertions he makes, or the conclusions he arrives at. He says: "I shall only allude here to those *known* to be injurious, premising, however, that future investigations may, in throwing more light upon the subject, determine that other fillings are inimical to health." The assumption here, that investigations already made have determined the injurious nature and action of any of the materials now used, as it seems to us, is not proven by any of his strictures on the means or methods at present practised. We quote from his text:

"The most universally used filling, excepting perhaps gold, is an amalgam consisting of about *two parts of tin, one part of silver*, and as much mercury as will cause the mass to adhere together. Actual experiments show that 0.12 grammes of the tin and silver mixed as above will require 0.09 grammes of mercury to form the cohesive mass used by dentists for filling teeth. When ordinary care is not exercised in the preparation of this material a much larger quantity of mercury would remain. The quantity of this amalgam inserted in a single tooth varies from 0.60 to 4.00 grammes, [10 to 60 grains,] and in the mouths of many individuals as much as 20.00 grammes, [300 grains] have been found inserted in the teeth. This amalgam, composed so largely of mercury, is usually much exposed to the attrition of mastication and the movements of the tongue and cheeks. The free mercury which it was found to contain by Dr. Wm. Stratford is worn off in small particles by the friction in the mouth. These particles, when submitted to dilute hydrochloride acid, yield a chloride of mercury. That toxic effects may resist from wearing these fillings in the teeth is, therefore, established."

*But is it established?* Certainly not from the statements or conclusions of Dr. Sexton. For, in the first place, the amalgams in common use are not composed of tin and silver mainly, but of other metals alloyed with these—not that there is anything detrimental to the constitution in this combination,—but that recent careful investigations have shown that other combinations of metals produce better results. Nor is it probable that, save in exceedingly exceptional cases, will amalgam fillings be found to weigh one-eighth of an ounce, as asserted by Dr S.

But it is to the statement that "toxic effects may result" from the attrition and loss of these fillings, and the chemical action by dilute hydrochloric acid, upon the particles thus worn away, that our attention is attracted. Dr. Sexton has well said, "*may* result;" for he will hardly find any-one at this day to state that such effects *will* result. It is perhaps, among the possibilities; certainly not among the probabilities. In the first place it is known that the old theory, so much harped on by the opposers of amalgam in the past, when neither its qualities nor capabilities had been at all carefully studied, that there remains "free mercury" in the filling, is untrue, except possibly where the grossest carelessness in manipulation exists. The combination between the finely divided metals and the mercury is exceedingly close, and only separable by the action of high temperature (nearly red heat,) or the action of acids so energetic as to attack the alloys themselves. Indeed the whole amalgamated mass may and should be considered an alloy, one part of which is as resistant to acids as another.

The assertion that there is loss of the substance of the filling by attrition, would be hardly made by one familiar with the subject. Even soft gold is with exceeding slowness worn away, when the opposing tooth grinds upon it; soft tin is known to last for years; and amalgams, at least those in ordinary use, are of all fillings the hardest. It could hardly be demonstrated, we think, that an ordinary amalgam filling would lose the one thousandth part of its weight in a year; and although Dr. Sexton quotes elsewhere in his paper to show that dilatation of the pupil has been caused by the four hundred and sixty thousandth part of a grain of atropia; even he would hardly assure us as of the toxic effect of the infinitesimal dose of chloride of mercury obtained from the attrition, etc., of an ordinary amalgam filling. The statement, in the same connection, that amalgam fillings have the effect to weaken the enamel, must be derived, as doubtless are the other supposed facts cited by Dr. Sexton, from the writings of the older opponents of amalagam, when from prejudice or antipathy, or a want of experimental knowledge of the facts in the matter, statements were made and left unrefuted, possibly until the present day.

Passing further, we notice the statement that when fillings are in the teeth and plates (metalic or vulcanite) are worn, the conditions for harm are yet more favorable. Our author has evidently here in mind the theory of "galvanic action," which, while it may not be without its claims to be heard, is, we are sure vastly overrated by those who advocate it. We have no disposition to begin an argument on the subject of the "New Departure," nor dispute the theories that there are galvanic currents between tooth substance and fillings, but it is hardly true that the presence of at least a vulcanite plate could complicate matters, in this direction at least.

We pass to the subject of plates with artificial teeth attached and their wearers, on which our author asserts that "it is believed that the health of a large number of these people is *imperiled by the material* used in the construction of plates, as well as the methods used in fitting them to the mouth;" and he claims that the amount of injury thus done is not likely to be known, from the fact that those injured do not recognize or are reticent about the injury. We have no defence to make of the practices alleged by Dr. S. to be in vogue, such as "plates put into the mouth over carious fangs, inflamed gums, and collections of tartar completely encasing them, and retaining the foul secretions and decomposed particles of food usually present." This is certainly very bad practice, and we hope that the number of dentists who are thus maltreating their patients is exceedingly small; though it may be well said in their defence that much of the blame attached to the dentists should be laid on the patients, who are notoriously careless of cleanliness.

Apparent stress is laid on the fact that in one of the cases cited by our author of detected disease, the dentist who made the diagnosis was also a physician; although the diagnosis in question could readily have been done by a student, and the unconsciousness of the patient himself to the abnormal condition of affairs is hardly credible. But it is with the greatest severity that Dr. Sexton attacks compounds of metals in the mouth, and he gives to the alleged fact that when a gold plate touches an amalgam filling, the latter is rapidly corroded, especial importance; and the same result he affirms ensues when amalgam and gold fillings touch each other. *This latter fact we have never been able to verify in a long practice*, nor have we ever met a practising dentist who could do so.

Vulcanite plates, however, are the subjects of the severest attack of all by Dr. Sexton, and to this we do not object, if it will enlighten the public mind upon some of the questions concerned with it on which at the present those interested seem in the dark. We have no defence to make of the "vile rubber," and question if dentistry has really gained by its introduction. But Dr. Sexton is not a "vulcanite dentist," and certainly has not studied the question in its real significance, else he would never have been betrayed into the errors which beset his article. After commenting upon the action of the fluids of the mouth upon low grades of metal plates, he states that when more than one kind of metal is used in their construction, this fact may cause chemical action, especially when gold touches amalagam, and he italicises these words: "*the well known fact that when two different metals are brought into contact in the presence of a dilute acid or solution of a salt, a current of electricity is generated*, should lead us to infer that their harmlessness to either the sick or well is problematical."

Problematical indeed! But the relation of cause and effect here, as elsewhere, is not known certainly, and until it is, it is hardly worth while to drag in, as does our author, the old theories of Burc, revived by Charcot and others, for the sake of illustrating so doubtful a subject. While people are subject daily to the action of agents ordinarily known as noxious or toxic, and become tolerant of them, even to the enjoyment of health, the infinitesimal dose of a nameless something begotten of an imperceptible and intangible galvanic action should hardly be the subject of so heavily an underlined paragraph. He may well say that "a further consideration of the subject would lead us too far."

In the next paragraph he puts down the vermilion in vulcanizable gum—that prepared for dentist's use—as equal to both the weight of the sulphur and caoutchouc. The text books give us as a formula: Caoutchouc 2, Sulphur 1, one and one-half part of this mixture and one part of Vermilion. Or Caoutchouc 48, Sulphur 24, Vermilion 36. Of course when the unvulcanized gum, thus prepared is "chewed for several hours, it yields a salt of mercury," as the mass has little cohesion, save the tenacity of the soft yielding gum. The question is vastly different when the compound is vulcanized.

"To bake this soft material into plates of sufficient hardness to support artificial teeth, a temperature of about 160° C. is required. *This degree of heat is not sufficient to completely volatilize the contained vermilion and sulphur.*"

Of course not! When the first substance is only separable from its compound association with sulphur at or about a red heat, and not volatile except when heated to decomposition, and if it were volatilizable, and this did occur, the result would be a *black plate*, (as rubber, minus vermilion, is black, or nearly so;) and the sulphur, instead of passing off by the heat, is combined with the gum, making a chemical union with it. Were it to be volatilized even in a small degree, the result would be a useless and unvulcanized plate, *as the presence of sulphur is absolutely necessary to the vulcanizing process.* Dr. Sexton evidently is not posted on the subject he is writing of.

Just here we quote, not for the benefit of Dr. S. alone, but for the many who find in vulcanite hidden and dangerous poisons, a paragraph from Makins, whose work on metallurgy is a standard one. He says: "Neither water, alcohol, the alkalies nor the mineral acids singly, have any effect upon vermilion." Dr. Sexton seems aware of the negative action of the acids in question, but charges the poisoning to the alkalies, or at least says, these break down the compound, and the vermilion thus liberated acts as a poison.

Further, the vulcanite plates besides "yielding a poison," are charged with injury to the health by reason of the imitation they produce; one-third of those wearing them, he claims finding them to produce this effect. This result is said doubtless justly to be attributable to the non-conducting properties of the plate. But when "*the close contact of these plates*" is made a cause of injury, the dentist will smile; as also when it is gravely stated that "the parts are often bathed in pus!" Of the cases cited by the Dr. we cannot dispute the facts, but it is odd that they occur in his part of the country only; but that a vulcanite plate worn in the mouth should "cause nausea, vomiting and purging, with stomach painful to the touch, the symptoms increasing in severity from day to day; the mouth swollen, sensitive, feverish," etc., with a slow recovery, followed by a cutaneous eruption lasting some weeks—all this is very curious, to say the least.

The only American author quoted, of a long list of authorities by Dr. Sexton, is Prof. Abbott. In conclusion we feel sure had Dr. Sexton allowed some one well posted on the subjects he has floundered amongst to have written up the part of his article we have thus attempted to review, it would have been better for the subject discussed. It is well to know however, what individual men are saying and thinking about our specialty, and possibly it may be well for them to know what we think of their cogitations. It is always bad to go out of one's line to discuss a subject; the liability to mis-state and fall into error becomes a certainty almost, and Dr. Sexton has proven himself no exception to the rule.

